# The Bacteriophages Therapy of Interdigital Pyoderma Complicated by Cellulitis with Antibiotic-Resistant *Pseudomonas aeruginosa* in a Dog—Case Report

**DOI:** 10.3390/vetsci10110642

**Published:** 2023-11-05

**Authors:** Mariana Grecu, Mădălina-Elena Henea, Cristina Mihaela Rîmbu, Cătălina Simion, Eusebiu-Viorel Şindilar, Gheorghe Solcan

**Affiliations:** 1 Department of Pharmacy, Faculty of Veterinary Medicine, Iași University of Life Sciences “Ion Ionescu de la Brad”, 8 Mihail Sadoveanu Alley, 700489 Iasi, Romania; mgrecu@uaiasi.ro (M.G.); madalina.henea@yahoo.com (M.-E.H.); 2 Department of Microbiology, Faculty of Veterinary Medicine, Iași University of Life Sciences “Ion Ionescu de la Brad”, 8 Mihail Sadoveanu Alley, 700489 Iasi, Romania; crimbu@uaiasi.ro; 3 Department of Surgery, Faculty of Veterinary Medicine, Iași University of Life Sciences “Ion Ionescu de la Brad”, 8 Mihail Sadoveanu Alley, 700489 Iasi, Romania; simion_catalina15@yahoo.com; 4 Department of Internal Medicine, Faculty of Veterinary Medicine, Iași University of Life Sciences “Ion Ionescu de la Brad”, 8 Mihail Sadoveanu Alley, 700489 Iasi, Romania

**Keywords:** bacteriophages, antimicrobials, antibiotic resistance, skin infection, *Pseudomonas aeruginosa*

## Abstract

**Simple Summary:**

In the context of increasingly serious bacterial infections that occur in animals, for which antibiotics no longer respond to therapy, the search of new alternatives is often of vital/major importance for saving patients. A polyvalent suspension of bacteriophages was chosen in this study as an alternative method to combat a skin infection in a dog that was shown to be refractory to antibiotics. Bacteriophage therapy showed significant results, proving their effectiveness in local treatment, as well as in preventing the systemic spread of infection with *Pseudomonas aeruginosa* in dogs.

**Abstract:**

*Pseudomonas aeruginosa* is a highly pathogenic bacterium with high pathogenicity, that can cause serious infections in all species and especially in dogs. Treatment of the infection induced by this bacterium can be a challenge considering that some strains have developed resistance to most classes of antimicrobials. The use of bacteriophages to alleviate infections caused by *Pseudomonas aeruginosa* has demonstrated their potential for both internal and external applications. This study aimed to illustrate the treatment with bacteriophages in bacterially complicated skin lesions that do not respond to antimicrobial therapy.

## 1. Introduction

Infections with *Pseudomonas aeruginosa* are common for humans and animals and occur after patient’s contact with pyocyanic bacillus. *P. aeruginosa* is a gram-negative bacterium, an opportunistic pathogen that usually lives in environments such as water and soil, animals easily coming into contact with this bacterium. In recent years, *P. aeruginosa* has been identified as one of the most resistant bacteria to antimicrobials in both humans and animals, especially pets [1]. Due to the significant increase in multidrug-resistant bacteria, bacteriophages represent one of the therapeutic alternatives for the prevention and especially for the treatment of bacterial infections in humans and animals [2,3,4,5].

Bacteriophage therapy for *P. aeruginosa* infections has been used for a very long time, but with the advent of various classes of antimicrobials, recognition of the benefits of bacteriophages has gradually decreased, and today, despite their effectiveness and safety, there is no known legislative form to approve their use in medical practice [6].

Bacterial resistance to most antibiotics has renewed interest in the use of bacteriophages. Bacteriophages represent one of the most abundant entities in the environment, and are widespread in nature, with the highest presence in aquatic environments [7]. They represent a group of small viruses that have the ability to invade different bacterial species, inducing either a lysogenic effect or a lysis effect on the bacteria. The lysis effect is the most important in therapy, by the fact that the bacteriophage multiplies inside the bacteria and releases new phage particles by lysing the host cell, thus leading to its death [8].

Bacteriophage therapy has been used in recent years because of the lack of therapeutic options for patients with infections that do not respond to antimicrobial therapy [9,10].

The concern regarding the health of pets has increased considerably in the last period, so an important emphasis has been placed on research, especially on finding new alternatives for the treatment of infections that do not respond to antibiotics. Recent studies offer a perspective to veterinarians on the possible acceptance of bacteriophage therapy in infections of pets and farm animals [11].

In this case report, we highlight the usefulness of bacteriophages in combating *P. aeruginosa* resistant to multiple antibiotics in a dog with interdigital pyoderma complicated with cellulitis.

## 2. Case Report

**Figure 1:**
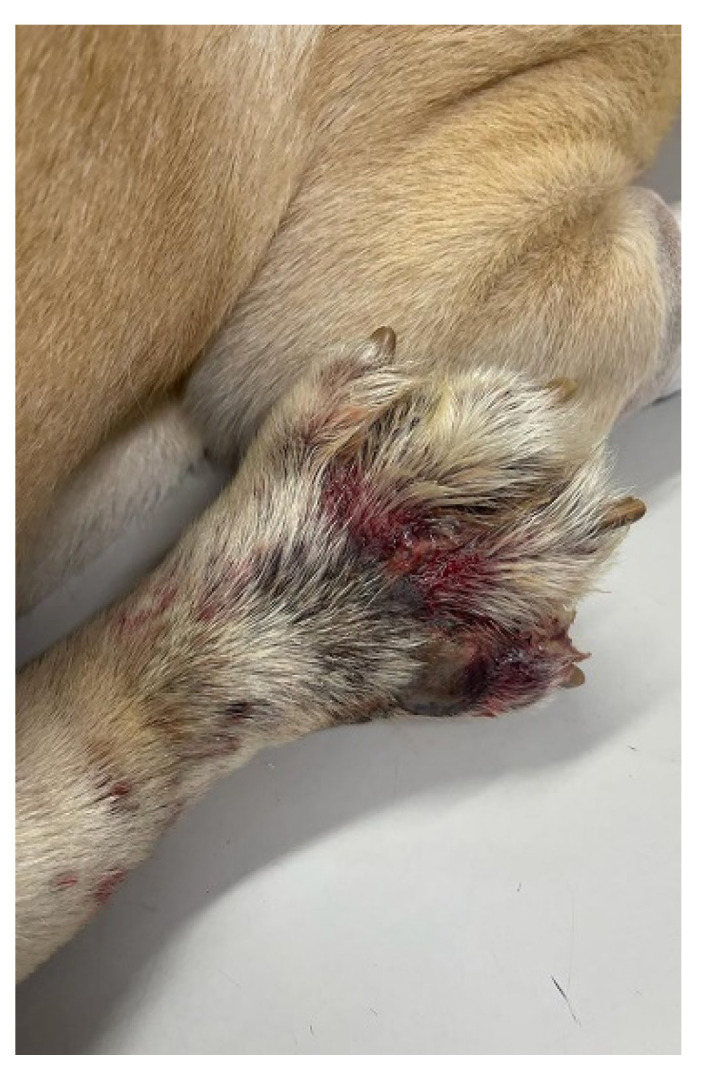
The clinical aspect at the first visit to the veterinarian; interdigital pyoderma complicated with cellulitis; the limb is oedematous expressing purulent exudate with traces of blood.

A male Labrador dog, aged 7 years and weighing 47 kg, was presented with an interdigital pyoderma on the right hind limb. The dog was intensely licking the affected area, and the limb was slightly oedematous and with traces of blood (Figure 1).

The dog was presented at the Surgery clinic of our faculty after previous visits to two private veterinary offices, and empirical antibiotic treatment. In the first clinic the lesion was disinfected with betadine and ceftriaxone was administered intravenously at a dose of 20 mg/kg every 12 h, and meloxicam was administered subcutaneously at a single dose of 0.2 mg/kg. The treatment was administered for 3 days, but the patient’s condition deteriorated rapidly under antibiotic therapy. The limb continued to swell, lameness, pain and avoidance of movement appeared, which led the owner to request the opinion of another veterinarian, in another office.

At the second private veterinary office, the dog was administered enrofloxacin 2.5 mg/kg, once every 24 h. After 2 other days of therapy, the dog’s general condition deteriorated completely. The dog had a rectal temperature of 39.1 °C, lack of appetite, dyspnoeic breathing, and the limb was swollen and hot, cyanotic, without the possibility of movement. Amputation of the affected limb was recommended because antibiotic therapy had no results and there was a risk of septic shock.

After the 5 days of empirical antibiotic treatment, the owner of the dog requested an appointment at the Surgery Clinic of the Faculty of Veterinary Medicine for the amputation of the affected limb. The surgeon recommended bacteriophage therapy instead.

A rapid onset of the infection was observed, manifested by inflammation, hardened tissue, swelling, the skin had a violet colour and small pustules that broke and highlighted the area of underlying necrosis. These phenomena were dominated by changes in the patient’s general condition including chills, tachycardia, dyspnoea, fever and agitation. The local erythema and oedema quickly spread up to approximately 20 cm from the location where the lesion initially appeared and affected the skin and connective tissue.

**Figure 2:**
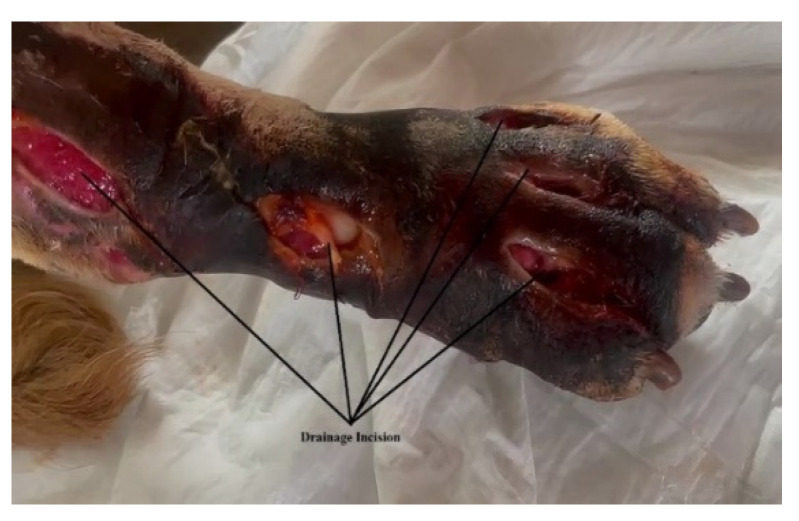
Application of drain incisions to eliminate the liquid collection; several drainage incisions were made to remove the purulent collection. The skin is necrotic and the limb strongly oedematous.

For diagnosis and treatment, the following stages of work were followed:

1. After the clinical examination, venous blood was collected and laboratory tests were performed, which included: blood count, C-reactive protein, fibrinogen, acute phase reactants (ESR), creatine phosphokinase (CPK), urea, creatinine, urine analysis, blood gas, electrolytes and glucose analysis. Cytological exam of the purulent exudate was made to rule out neoplasia, fungal and parasitical infection.
2. Imaging investigations were carried out with computed tomography (CT), to highlight the presence of subcutaneous air that frequently occurs in the case of infections, but also to observe areas of muscle necrosis and to reveal a possible foreign body.
3. Drain incisions were made in order to eliminate the liquid from the edema level (Figure 2) and samples were taken from the liquid for bacteriological cultures, as well as samples of tissue fragments from the necrotic area. The work steps were performed according to conventional methods of clinical microbial analysis. The samples were plated on regular culture media (Mueller Hinton blood agar, Oxoid) and incubated at 37 °C. After 24 h, the cultural, morphological and biochemical aspects of the bacterial strains that grew on the surface of the culture medium were examined.

4. An antibiogram was performed in order to determine the use of systemic antibiotics. Strains isolated from the pure culture were tested against different panels of antibiotics. Antimicrobial susceptibility testing of the isolated strains was performed using the Kirby-Bauer disk diffusion method according to international standards (EUCAST, 2023). The evaluation of the results was based on the diameter of the inhibition zones determined by the antibiotics: Norfloxacin (10 µg), Doxycycline (30 µg), Enrofloxacin (30 µg), Ampicillin (10 µg), Amoxicillin-Clavulanic Acid (10 µg), Neomycin (30 µg), Ceftiofur (30 µg), Ceftriaxone (30 µg, Oxoid). Based on the results obtained, the strains were classified as “S” susceptible and “R” resistant [12].
5. The dog was subjected to supportive therapy with infusion solutions of sodium chloride, Ringer and glucose 5%.
6. The necrotic area was periodically cleaned and disinfected with hypochlorous acid (HOCl), a potent antibacterial and antiviral agent [13], for two days, until the results of bacteriological examination and antibiogram were obtained. After delimiting the necrotic areas, the bacteriophage treatment was initiated according to the manufacturer’s instructions. The commercial suspension used in therapy contains a mixture of sterile purified phagolyzed filtrates from 6 types of microorganisms (Piobacteriophage Polyvalent Polyphage—Sixtaphages, MIKROGEN, Russia) and contains bacteriophages for *Staphylococcus* spp., *Streptococcus* spp., *Proteus vulgaris*, *Proteus mirabilis*, *Pseudomonas aeruginosa* and enteropathogenic *Escherichia coli*.
7. For each application, 40 mL of bacteriophage suspension was used in the form of swabs on the injured surface, and the therapy was applied intermittently for a period of 3 months. The bacteriophage suspension was administered 2 times/day, at 12-h intervals for a period of 20 days. After this period, the suspension was administered once a day for 10 days. Bacteriophages were then applied every three days, for 60 days (as a preventive measure against microbial reinfection), until complete healing of the lesion of the affected limb was obtained. After using bacteriophages and leaving the treated area for a few minutes, a bandage impregnated with neutral ointment and paraffin (Grassolind^®^, Hartmann) was applied, in order to stimulate the formation of granulation tissue, epithelization, and prevent adhesion of the wound’s bandage.

The study was carried out with the consent of the Ethics Commission of the Faculty of Veterinary Medicine Iași, within the “Ion Ionescu de la Brad” University of Life Sciences in Iași, in accordance with the Research Law no. 206/27.05.2004 on good practices in scientific research, technological development and innovation, as well as European Legislation. The owner of the dog provided written informed consent for the publication of this case report and accompanying images of the progression of bacteriophage therapy.

**Figure 3:**
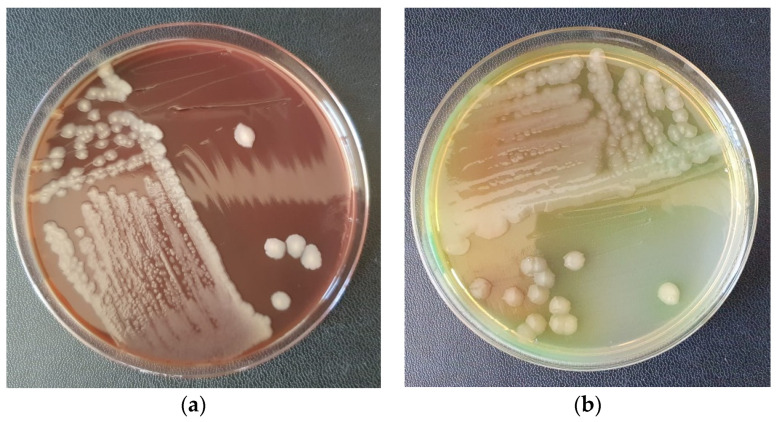
Pure culture of *Pseudomonas aeruginosa* from the collected samples; (**a**) Seeding of pathological products on Mueller Hinton Agar with 5% Sheep Blood (Oxoid). (**b**) Inoculation of pathological products on Mueller Hinton Agar (Oxoid).

Microbiological examination of the exudate obtained from the incision and of the tissue fragments taken from the necrotic area revealed a pure culture of *Pseudomonas aeruginosa* (Figure 3a,b). 

Laboratory examinations of blood samples did not show any changes in the evaluated parameters and did not demonstrate the existence of a systemic infection. Creatinine phosphokinase was between the normal values (1.2 mg/dL), indicating that the infection did not lead to muscular tissue necrosis. At the previous clinical examination and throughout the treatment period, the dog was normotensive and normoglycemic.

CT did not show any foreign body and did not highlight muscle necrosis or air in the subcutaneous space, but a moderate thickening of the subcutaneous tissue and fascial areas in the lesion area was observed.

**Figure 4:**
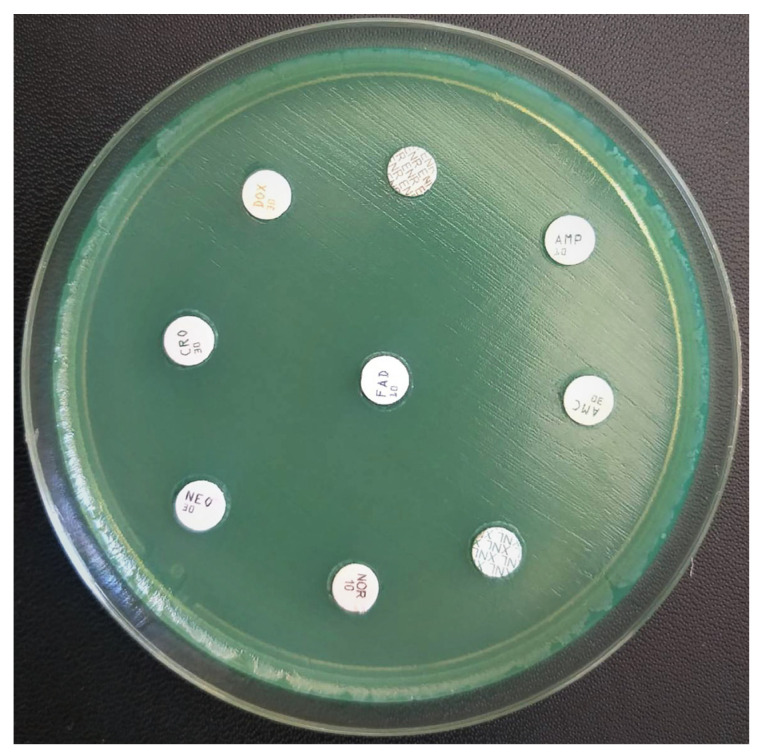
Antibiogram of *P. aeruginosa* on Mueller-Hinton agar, proving antibiotic resistance.

The antibiogram performed on the exudate obtained from the incisions showed the resistance of *P. aeruginosa* to antibiotics usually used in veterinary medicine (Figure 4).

**Figure 5:**
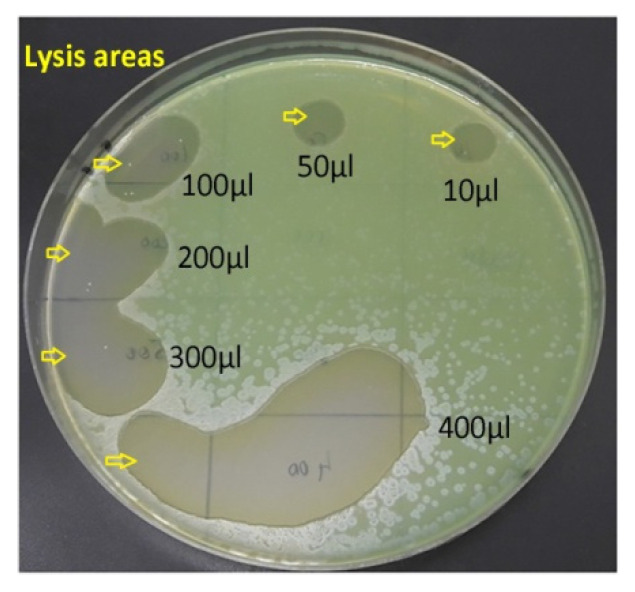
Bacterial lysis induced by bacteriophages: areas without bacterial culture (lysis zones) indicate that the phages have killed the *P. aeruginosa* cells (yellow arrows). The lytic effect was directly proportional to the number of bacteriophages present in the suspension.

Figure 5 shows the cultural appearance of lawn-grown *P. aeruginosa* strain on Mueller Hinton Agar (Oxoid) onto which various amounts of bacteriophages (10 µL, 50 µL, 100 µL, 200 µL, 300 µL, 400 µL) were spotted with an automated pipette. 1 mL of the suspension contains <1 × 10^5^ bacteriophages. 

Supportive treatment was completed by surgical treatment to remove the necrotic tissue in order to stop the spread of the infection.

**Figure 6:**
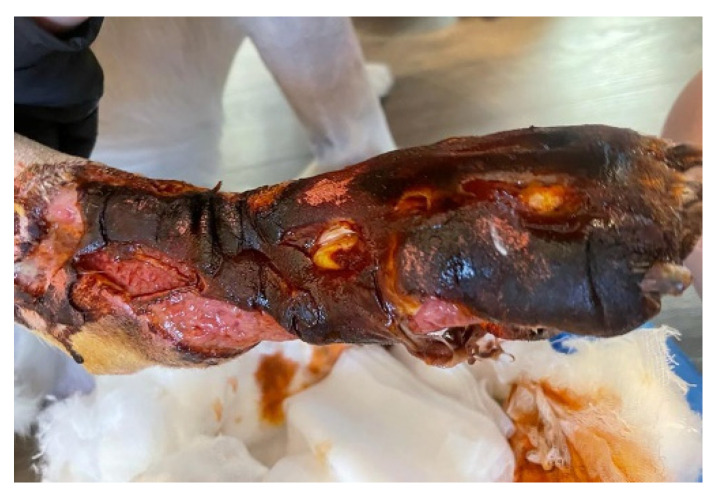
Debridement of necrotic tissues; wide incisions were made with debridement and excision of all necrotic areas—cellular tissue, fascia, skin—over the entire extent of the skin detachment. The skin was excised only on the necrotic areas, protecting the muscles.

Gradually, the necrotic areas began to be delimited within approximately 2 weeks, and the necrotic area was debrided daily, by excision of the devitalized tissues (Figure 6).

**Figure 7:**
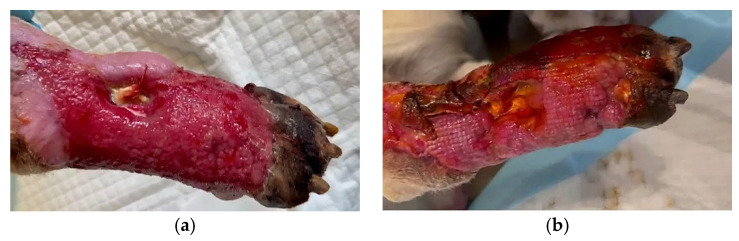
Application of bacteriophages on the lesion, under protection with a bandage. (**a**) before the application of bacteriophages solution. Soft tissue destruction to bone is observed. (**b**) after the application of bacteriophages solution.

When the wound was dry and the necrotic areas were well-defined, the bacteriophage solution was applied by swabbing directly on the lesion, intermittently, for a period of 90 days (Figure 7).

**Figure 8:**
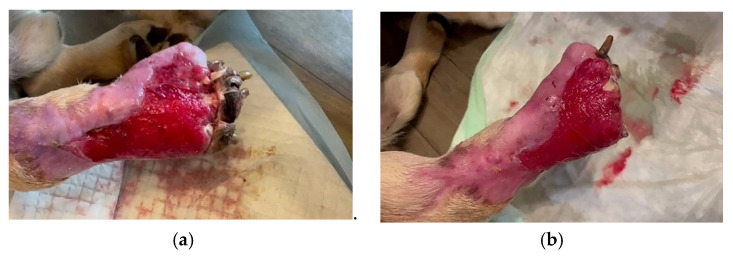
Wound aspect after bacteriophage therapy; (**a**) The aspect of the lesion 30 days after the application of the bacteriophage solution. The lesion is reduced, granulation occurs and the ulcer progresses continuously and evolves towards healing. (**b**) A new surgical intervention for the excision of necrotic fingers.

With each use of bacteriophages, improvements were observed in the lesion with the presence of vivid red granulation tissue, indicating the evolution towards healing, without the occurrence of necrosis (Figure 8a). In order to avoid re-infection of the lesion, approximately 30 days later surgical intervention was performed to excise the necrotic fingers. The wound was active and bleeding, which suggests the healing process (Figure 8b).

**Figure 9:**
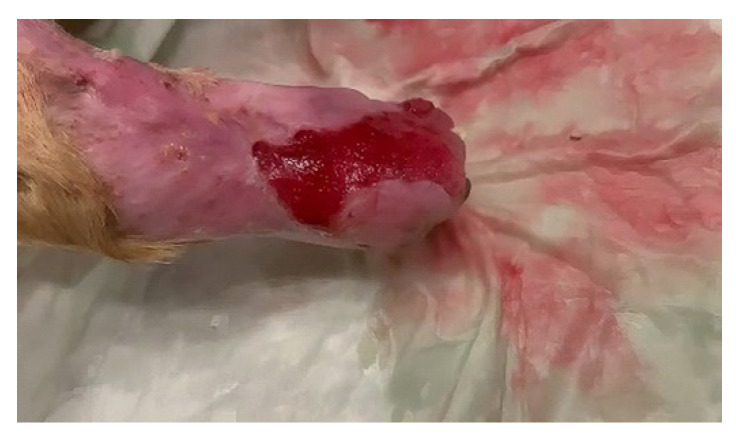
Evolution of the lesion. After 60 days of treatment, the initial outline of the ulcer is over 70% epithelialized, the evolution being favorable.

The healing occurred with epithelization from the periphery, and the ulceration was covered to a large extent with granulation tissue formed by neoformation of vascular buds (Figure 9).

**Figure 10:**
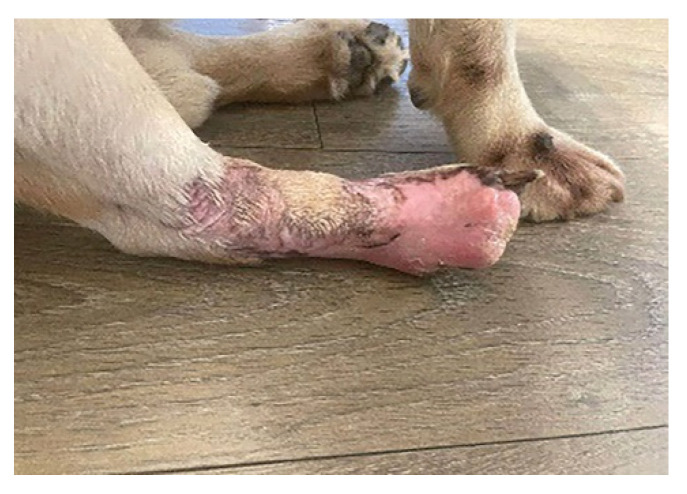
Complete epithelization of the skin lesion at the level of the limb.

The treatment ended when the necrotic area was completely epithelialized, and the entire recovery process took place over a period of 3 months (91 days). During the entire period of bacteriophage therapy, gradual remodeling and maturation of the scar was observed, with gradual reduction of the continuity solution until disappearance. Exuberant granulation tissue, which often occurs in the case of large lesions, did not appear, but a clean scarring occurred, with a physiological epithelization, and the patient resumed his normal daily activity (Figure 10).

The study highlighted that bacteriophages induced a high rate of healing of the skin lesion and accelerated the skin remodelling process. Thus, phage therapy proved effective for the treatment of bacterial infection with *P. aeruginosa*, which was resistant to antibiotics commonly used in veterinary medicine.

## 3. Discussion

Etiologic factors of interdigital pyoderma include foreign bodies (e.g., foxtails, awns, thorns, wood slivers, seeds) and local trauma. When a single foot is affected, foreign body, local injury, or neoplasia should be suspected, especially if there is only one interdigital fistula. Fungal infections associated with pododermatitis include dermatophytosis, *Malassezia* dermatitis, candidiasis, mycetoma, phaeohyphomycosis, sporotrichosis, blastomycosis, and cryptococcosis. Even though these are uncommon, they should be suspected in cases that are refractory to usual antibiotic therapy. Bacterial infections are always secondary and can include a wide variety of organisms. Cases of chronic interdigital pyoderma must be evaluated carefully for demodectic mites and other parasites that may be involved: *Pelodera strongyloides*, *Ancylostoma* spp., and *Uncinaria stenocephala*. Despite multiple etiologies, a substantial number of cases are idiopathic recurrent bacterial infections [14].

Antimicrobial resistance is one of the most pressing public health problems for the future, and the veterinary profession needs to be proactive in monitoring and controlling antibiotic resistance and the use of antimicrobials in small animals at the local, national and international levels. The prudent, strategic use of antimicrobials, reserving systemic application for deeper and more complicated infections, may limit the spread of multi-drug resistant bacteria in patients with skin disease [15]. A rapid global spread of methicillin-resistant *Staphylococcus pseudintermedius* (MRSP) clones displaying multi-drug resistance in dogs was observed [16]. Antimicrobial resistance patterns of *S. intermedius* and *S. pseudintermedius* isolated from dogs and cats appear to vary with their geographical origin because of different approaches to antimicrobial use in companion animals [15,17].

*P. aeruginosa* is one of the most important strains of its species in veterinary medicine and infections caused by this agent are increasingly common in animals. Bacteria get in the subcutaneous tissue through a small lesion, the tissue providing a favourable environment for bacterial development. As bacteria multiply, they produce toxins and proteases that cause local tissue damage. Toxic shock syndrome can occur as a result of tissue destruction and hematogenous spread of endotoxins and inflammatory mediators. In this study, tissue destruction had an extremely rapid progression and was extended over a large surface of the limb by the exponential multiplication of *P. aeruginosa*, a bacterium that was evident in the pure culture.

*P. aeruginosa* has the extraordinary ability to build resistance through multiple mechanisms, often simultaneously, resulting in resistance to nearly all available antibiotics. The main resistance mechanisms in *P. aeruginosa* are often divided into intrinsic and acquired, which counteract most antibiotics [18,19].

The results of this report showed that the strain of *P. aeruginosa* showed multiple resistance to aminopenicillins + beta-lactamase inhibitors (amoxicillin-clavulanic acid), penicillins (ampicillin), aminoglycosides (neomycin), fluoroquinolones (enrofloxacin, norfloxacin), tetracyclines and third-generation cephalosporins (ceftriaxone). Given that infections produced by *P. aeruginosa* have a rapid progression and a high mortality rate, especially in dogs, rapid intervention is necessary through early and complete surgical debridement, systemic support and antibiotic therapy. However, the increasing number of reports describing the resistance of bacterial infections to antibiotics encourages the use of new therapeutic approaches [19,20]. One of the therapeutic options for patients with infections refractory to antibiotic therapy is based on bacteriophages. These are a diverse collection of viruses that are easy to use, in various antibacterial treatments [2,3,4,5,6,21].

In veterinary medicine, studies of bacteriophage therapy to combat skin infections in pets are limited, however bacteriophages have been proved to have potential in the treatment of antibiotic-resistant infection with *Pseudomonas aeruginosa* [22,23].

Considering the rapid evolution of the patient’s infection in this study, with no chance of recovery due to the extensive antimicrobial resistance of the *Pseudomonas aeruginosa* species, an attempt was made to optimize the treatment with a bacteriophage suspension. The treatment with bacteriophages was well tolerated by the patient throughout the administration, without any side effects. The clinical condition improved continuously, along with the gradual healing of the skin lesion.

Some clinical studies have provided encouraging results for the use of bacteriophages in infectious otitis in human patients [24] and in dogs [22]. Other studies have reported the benefits of bacteriophages in the treatment of experimentally induced systemic infections in animals, especially against experimental biofilms [25,26]. The remarkable ability of phages to adapt to bacterial defense systems has grown enormously. This adaptability has manifested in several active forms, such as the evasion of bacterial CRISPR–Cas (clustered regularly interspaced short palindromic repeats–CRISPR-associated proteins) immunity using anti-CRISPR proteins [4]. Five distinct ‘anti-CRISPR’ genes were found in the genomes of bacteriophages infecting *P. aeruginosa*. The existence of anti-CRISPR genes presents new paths for the elucidation of CRISPR/Cas functional mechanisms and provides new insight into the co-evolution of phages and bacteria [27].

Several studies in both human and veterinary medicine have demonstrated the potential of bacteriophage therapy for systemic, localized or cutaneous bacterial infections. However, the applicability of this therapy has not been fully demonstrated in clinical trials. Phage therapy has many advantages for veterinary medicine however, further well-conducted studies are needed to define the role and safety of phage therapy in daily clinical practice for treating animals with various infections.

## 4. Conclusions

The emergence of multidrug-resistant species of *P. aeruginosa* represents a zoonotic risk and contributes to increased morbidity and mortality in both human and veterinary medicine. This case study highlights the beneficial effect of the interaction between bacteria and bacteriophages in topical therapy, i.e., the particular ability to lyse, in vivo, the bacterial biofilm produces by *P. aeruginosa* in deep skin infection. Using mixed bacteriophages with distinct but overlapping specificity profiles has the benefit of lowering the possibility of developing bacteriophage-resistant bacterial strains that can emerge during veterinary or medical use and potentially pass from treated animals to humans. The study opens up new possibilities for extending research into the use of bacteriophages to optimize the use of antimicrobials in infections with antibiotic-resistant microorganisms.

## Data Availability

The data present in this study are available within the article.
